# Current status of various types of ICG contrast imaging in pediatric thoracic surgery

**DOI:** 10.1007/s00595-026-03411-z

**Published:** 2026-07-15

**Authors:** Shinya Takazawa, Ken-Ichiro Konishi, Michimasa Fujiogi, Jun Fujishiro

**Affiliations:** 1 Department of Pediatric Surgery, The University of Tokyo Hospital, 7-3-1 Hongo, Bunkyo-ku, Tokyo, 113-8655 Japan; 2 Department of Pediatric Surgery, Japanese Red Cross Medical Center, 4-1-22 Hiroo, Shibuya-ku, Tokyo, 150-8935 Japan

**Keywords:** ICG contrast imaging, Thoracic surgery, Pediatric surgery

## Abstract

Indocyanine green (ICG) fluorescence imaging is an emerging intraoperative navigation tool. However, its application in pediatric thoracic surgery remains to be elucidated. This review evaluates the current status and clinical utility of ICG-based imaging techniques in the field. A narrative review of studies published between January 2015 and February 2026 was conducted using PubMed, Web of Science, and Cochrane Library. Relevant original articles, reviews, and case reports were included. Data were analyzed by focusing on indications, administration methods, and clinical outcomes. Additionally, representative cases of chylothorax and congenital pulmonary airway malformations from our institution were included. Thirty-eight studies were included in this meta-analysis. ICG imaging enables real-time visualization of lymphatic leakage in chylothorax, rapid identification of intersegmental planes in pulmonary resections, and ventilation-based lesion delineation using inhaled ICG. It also supports fistula localization and perfusion assessment in patients with esophageal diseases. In oncology, ICG shows high sensitivity for detecting hepatoblastoma lung metastases, although its specificity is limited. ICG fluorescence imaging improves intraoperative visualization and precision during pediatric thoracic surgery. However, current evidence remains limited, and further standardization and prospective validation are required.

## Introduction

Indocyanine green (ICG) is a water-soluble tricarbocyanine dye developed in 1955 [1] and approved for clinical use in 1956 [2]. It absorbs and emits light in the near-infrared (NIR) spectrum, allowing real-time visualization of vascular and tissue structures during surgery. After intravenous administration, ICG rapidly binds to plasma proteins and remains largely confined to the intravascular compartment. It is selectively taken up by hepatocytes and excreted into the bile with a short half-life of approximately 2–4 min, permitting repeated intraoperative injections [3–5]. Its fluorescence penetrates tissues to a depth of 10–15 mm and has an excellent safety profile; however, caution is required in patients with iodine hypersensitivity [5–8].

Initially, ICG was used for diagnostic purposes, including the measurement of cardiac output and assessment of the hepatic function [9]. Its adoption in retinal angiography demonstrated the feasibility of fluorescence-based vascular imaging and laid the foundation for fluorescence-guided surgery (FGS) [5]. With advances in NIR camera systems and minimally invasive surgical platforms, ICG has been integrated into adult surgical practices. Following its first reported use in pediatric cardiac surgery in 2009 [10], ICG has been applied across multiple pediatric subspecialties [11]. Although many studies have been published on fluorescence-guided surgery using ICG in pediatric surgery, the number of cases is smaller than that in adult surgery, resulting in a limited number of papers with high levels of evidence. Most pediatric studies remain descriptive and limited in scale, and standardized protocols have not yet been established.

Although numerous review articles summarizing these papers have been published in recent years, no comprehensive review of pediatric thoracic surgery has been conducted. A review published in 2024 by Szavay et al. [12] broadly investigated fluorescence-guided surgery using ICG in pediatric surgery; however, only 16 papers were cited for the thoracic region, and details were mainly provided for metastatic lung tumors. A 2025 report by Di Mitri et al. [11] also broadly summarized the dosage and timing of fluorescence-guided surgery using ICG in pediatric surgery, but only chylothorax and metastatic lung tumors were mentioned in the thoracic region. Several similar reviews have been published, some focusing on neonates [13], others on oncology [14–16], and others providing broad coverage without specific dosage information [5, 8]. Therefore, this narrative review focuses on the application of ICG fluorescence imaging in pediatric thoracic surgery, an area in which consolidated reviews are limited.

## Methods

This narrative review aimed to broadly collect and understand the current status of ICG fluorescence use in pediatric thoracic surgery. Literature was collected from January 2009 to February 2026 using PubMed, Web of Science, and Cochrane Library. The following search formula was used: (“indocyanine green” OR “ICG”) AND (“Thoracoscopy” OR “Thoracoscopic surgery” OR “Thoracic surgery”) AND (“pediatric” OR “infant” OR “neonate”). As a limited number of articles were anticipated, the review included original articles, review articles, and case reports.

The collected articles were evaluated by three reviewers (ST, KK, and MF) and described for each disease, focusing on the use of ICG, sites of fluorescence, and clinical effects. Furthermore, we introduce the ICG contrast-enhanced imaging techniques and actual case examples used in our department for chylothorax and congenital pulmonary airway malformation (CPAM).

## Results

**Fig. 1:**
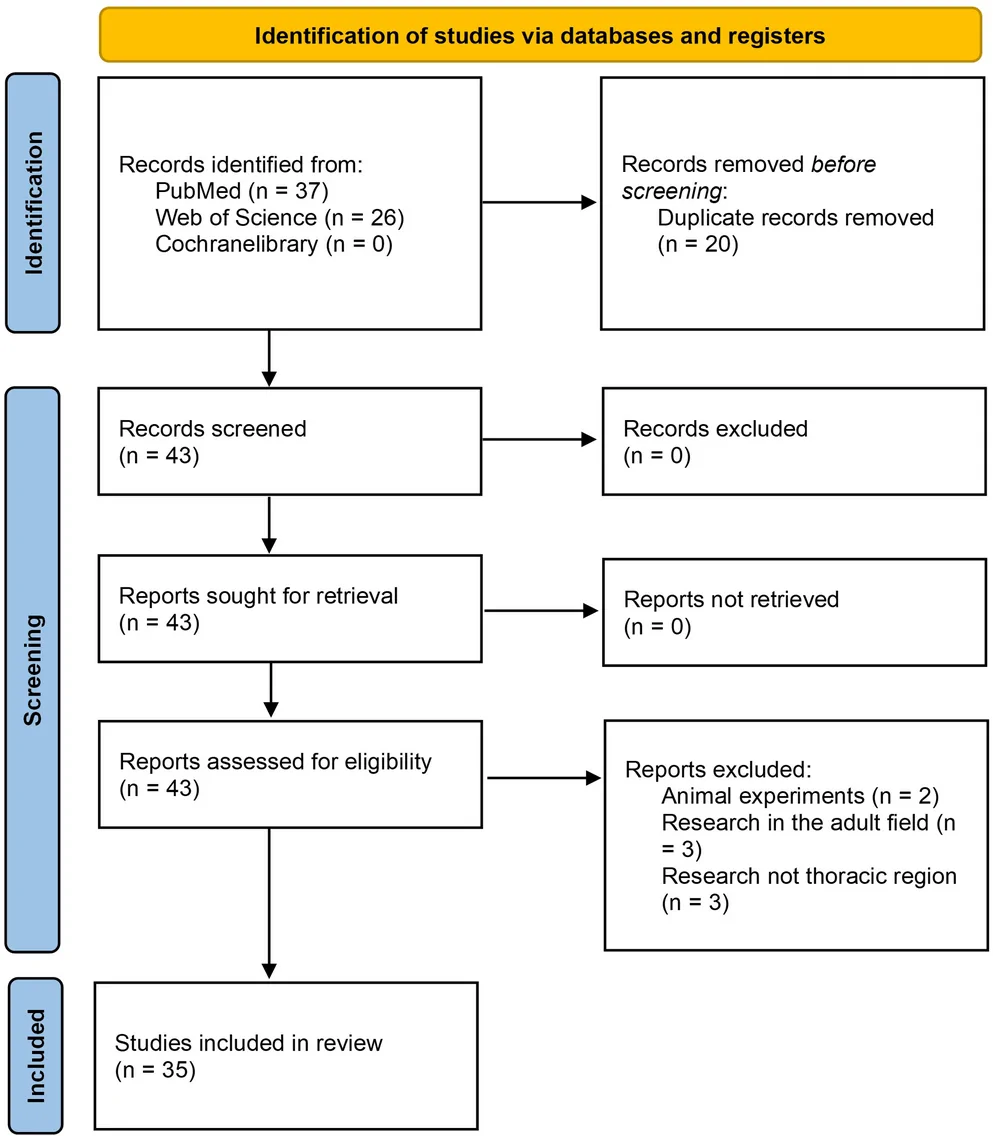
PRISMA flow diagram

The initial literature search yielded 63 relevant articles. As shown in Figs. 1 and 35 articles were reviewed. The reviewed articles included 19 original articles, 10 case reports, and six reviews. The review results for each disease are described below, and the typical ICG doses, timing, and imaging sites are summarized in Tables 1 and 2, respectively.

**Table 1:** Indocyanine Green (ICG) Usage in Pediatric Thoracic Surgery without Oncology

Category	References	Indication	Dose	Timing	Visualized Structure
Chylothorax	Takada et al. [17]	Leak/duct	0.1 mg/kg (toe)	Pre-op	Leak site
	Shirotsuki et al. [18]	Leak/duct	0.025 mg/body (toe)	1 h pre-op	Thoracic duct Leak site
	De Pauw et al. [19]	Exploration	NR	Intra-op	Thoracic duct
	Our case	Leak/duct	0.2 mg/kg (lymph duct)	Pre-op	Leak site
Lung (Intravenous)	Huang et al. [20]	CPAM	0.25 mg/kg	After vessel ligation	Segment plane
	Our case	CPAM	0.25 mg/kg	After vessel ligation	Segment plane
	Huang et al. [21]	CPAM	0.15 mg/kg	After vessel ligation	Segment plane
	Takenouchi et al. [22]	Sequestration	0.1 mg/kg	After aberrant vessel ligation	Boundary
	Nishida et al. [23]	Bronchial Atresia	0.6 mg/kg	Intra-op	Vessels
Lung (Inhaled)	He et al. [24]	CPAM	0.25 mg/kg	1–2 h pre-op	Normal lung
	Yin et al. [25]	Sequestration	0.5 mg/kg	30 min pre-op	Normal lung
	Zhu et al. [26]	CPAM	0.5 mg/kg	30–60 min pre-op	Normal lung
Esophagus	Castrillo et al. [13]	TEF	~0.15 mg/kg (Gastrostomy, iv)	Intra-op	Distal Pouch, Perfusion
	Zhao et al. [27]	TEF	0.25 ml (Broncho/ Esophagoscopy)	Pre-op	Fistula, Proximal Pouch
	Onishi et al. [28]	LGEA	0.5 mg/kg (iv)	Post-anastomosis	Perfusion
	Tsuruno et al. [29]	TEF	0.5 mg/kg (iv)	Post-anastomosis	Perfusion
	Guo et al. [30]	H-TEF	0.1–0.3 mL (Bronchoscopy)	Pre-op	Fistula
	Hua et al. [31]	Recurrent TEF	0.5 mL (Broncho/ Esophagoscopy)	Pre-op	Fistula
PPC	Mori et al. [32]	PPC	0.5 mg/kg/L	Intraperitoneal	Fistula

**Table 2:** Indocyanine Green (ICG) Usage in Thoracic Pediatric Oncology

Category+B3:G20	References	Pathology	Dose	Timing	Visualized Structure
Pulmonary metastases	Lake et al. [33]	Hepatoblastoma	0.5 mg/kg	24 h pre-op	Tumor fluorescence
	Delgado-Miguel et al. [34]				
	Kitagawa et al. [35]				
	Yamamichi et al. [36]				
	Whitlock et al. [37]	Hepatoblastoma	0.2–0.75 mg/kg	24–96 h pre-op	Tumor fluorescence
	Jeremiasse et al. [38]	Osteosarcoma	0.5–1.0 mg/kg	24 h pre-op	Tumor fluorescence
	Kodikara et al. [39]	Osteosarcoma	4.5 mg/kg	24 h pre-op	Tumor fluorescence
	Gallego et al. [40]	Ewing sarcoma	0.2 mg/kg (Inhaled)	Immediately pre-op	Negative contrast
	Harris et al. [41]	Ewing sarcoma, etc.	0.5 mL (Bronchoscopy)	Pre-op	Tattoo of inject site
Chest wall/ Diaphragm recurrence	Whitlock et al. [37]	Hepatoblastoma	0.2–0.75 mg/kg	48–96 h pre-op	Tumor fluorescence
Mediastinal tumor	Nakamura et al. [42]	NSGCT	~12.5 mg	Intra-op	Phrenic nerve
Sentinel lymph node mapping	Schermerhorn et al. [43]	Melanoma, Scc, etc.	4 guadrants around tumor	Pre-op	Lymph nodes
	Johnston et al. [44]	Sarcoma, Scc, etc.	0.3 mg	Pre-op	Lymph nodes
			x 4 guadrants around tumor		
	Campwala et al. [45]	Melanoma, Sarcoma, etc.	0.5-1 mg	10–20 min pre-op	Lymph nodes
			x 4 guadrants around tumor		

### Chylothorax (identification of leakage site and thoracic duct)

Chylothorax is defined as the accumulation of chyle within the pleural cavity due to leakage from the thoracic duct or its tributaries. In recent years, near-infrared fluorescence lymphangiography using ICG has emerged as a valuable modality for real-time visualization of the thoracic duct and leakage sites. Following subcutaneous injection into the interdigital space of the toes, ICG travels along the lymphatic flow pathway, enabling the fluorescent visualization of the thoracic duct and potential leakage points. Takada et al. [17] administered 0.25 mg (approximately 0.1 mg/kg) of ICG subcutaneously into the interdigital toe space and successfully identified and ligated the leakage points, achieving a 90% resolution rate within three weeks.

Similarly, Shirotsuki et al. [18] reported that in neonatal cases, ICG injected into the interdigital toe space one hour prior to surgery allowed direct visualization of the thoracic duct and leakage points under near-infrared imaging. This technique has proven particularly beneficial in reoperative cases and when dealing with microscopic leaks that are otherwise difficult to detect.

However, De Pauw et al. [19] described a case in which the thoracic duct was visualized using ICG, but no active leakage site was identified, suggesting that although ICG lymphangiography is highly useful, it may not universally detect all leakage points.

**Fig. 2:**
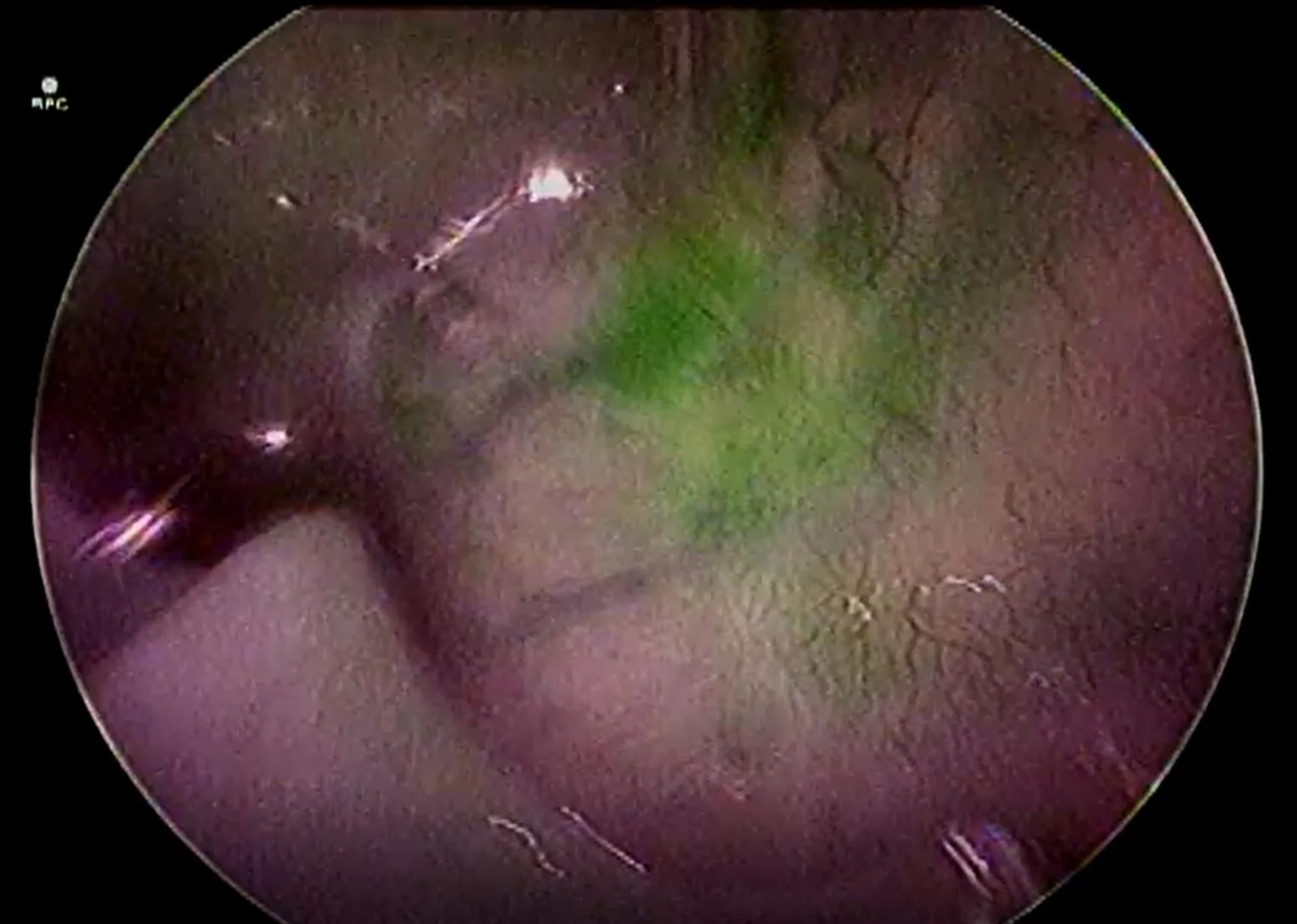
After injection of 2 mg/kg ICG thorough right inguinal lymph duct, bright areas were detected near the ligation site of thoracic duct under thoracoscopy

In our experience, ICG lymphangiography can detect lymphatic leakage points during thoracoscopy, even in a 6-month-old patient who underwent thoracic duct ligation. First, the right inguinal lymph duct was cannulated using a 27G needle, and 2 mg/kg ICG was injected under microscopic surgery. The intrathoracic cavity was carefully observed under thoracoscopy, and bright areas were detected near the ligation point of the thoracic duct and venous angle (Fig. 2). The bright areas were coagulated using a monopolar solution, covered with Polyglycolic Acid Sheets, and sprayed with BOLHEAL. The right chylothorax decreased in size immediately after surgery.

The principal advantages of ICG lymphangiography are real-time visualization, adaptability to anatomical variations, and minimal invasiveness. Owing to these characteristics, this technique is becoming an increasingly essential adjunct in the surgical management of refractory pediatric chylothorax.

### Intravenous ICG for lung segmentectomy

Precise identification of the intersegmental plane (ISP) is critical during segmentectomy for CPAM, pulmonary sequestration, and bronchial atresia. Compared with the conventional inflation–deflation method, intravenous ICG-guided near-infrared fluorescence imaging provides rapid and clear visualization of ISP.

Huang et al. [20] reported that after isolating and transecting segmental vessels, the intravenous administration of ICG at 0.25 mg/kg enabled ISP visualization within 5–10 s. The perfused lung segments fluoresced, whereas the target segment with interrupted blood flow remained unstained, allowing for clear demarcation. In their cohort, the time required to visualize the ISP was markedly shorter than that required for the modified inflation–deflation method, and the overall operative time was significantly reduced.

**Fig. 3:**
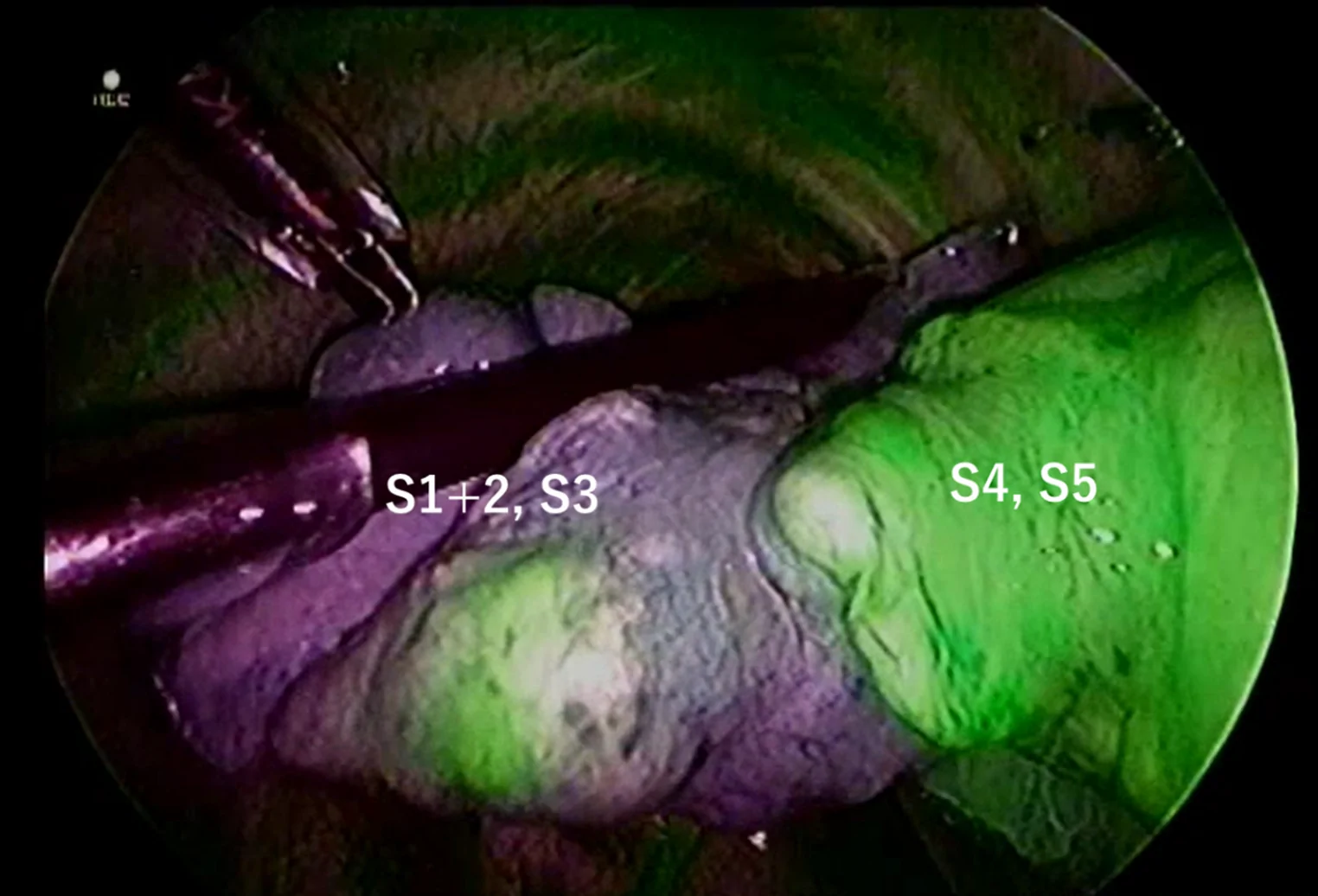
After ligation of A1+2 and A3, ICG (0.25 mg/kg) was administered intravenously, and the intersegmental plane between the superior lung segments (S1+2 and S3) and the lingular lung segments (S4 and S5) was clearly identified

In our experience, ICG-guided thoracoscopic multilobar resection is effective for congenital pulmonary airway malformations with abnormal segments and lesions distributed across multiple lung lobes (S1 + 2, S3, and S6). After vascular ligation, ICG (0.25 mg/kg) was intravenously administered, and resection was performed successfully after confirming the unstained areas (Fig. 3).

Huang et al. [21] reported that a lower dose (0.15 mg/kg) was sufficient for accurate ISP delineation without compromising the safety of the patient.

Takenouchi et al. [22] reported that intravenous administration of 0.1 mg/kg ICG after ligation of aberrant vessels resulted in fluorescence of normal lung tissue, while the sequestrated lung remained non-fluorescent, thereby enabling precise identification of the resection margins.

The mechanism underlying this method is perfusion-dependent contrast. After the segmental vessels were divided, ICG was distributed only to the perfused lung tissue. Consequently, the area designated for resection, where blood flow was interrupted, remained non-fluorescent. This perfusion-based contrast mechanism allows for the accurate visualization of the intersegmental boundary. This technique is particularly valuable in infants, in whom the risk of misdiagnosis is high owing to their small lung size, small body size, and difficulty in using an air-filled collapse line.

### Inhaled or intratracheal ICG for CPAM resection

A more recent development involves the use of inhaled (nebulized) ICG to delineate lesion boundaries based on differences in ventilation rather than perfusion.

He et al. [24] administered nebulized ICG at a dose of 0.25 mg/kg 1–2 h before surgery. Under fluorescence imaging, normal lung tissue exhibits uniform green fluorescence, whereas CPAM lesions, due to impaired ventilation, show little or no fluorescence, creating a distinct demarcation. Subsequently, lesions that did not fluoresce with ICG were excised using a clip-and-vessel sealing system, and good results were obtained.

In a multicenter study, Yin et al. [25] used a higher inhaled dose (0.5 mg/kg) and demonstrated that the technique was safe and associated with shorter postoperative drainage duration, reduced hospital stay, and improved postoperative pulmonary function compared with conventional techniques.

Similarly, Zhu et al. [26] administered 0.5 mg/kg of aerosolized ICG 30–60 min preoperatively and achieved clear visualization of the intraoperative lesion boundary.

The mechanism of this approach is ventilation-dependent contrast. In normal lung tissue with preserved ventilation, the inhaled ICG is distributed in the alveoli and fluoresces under UV light. In contrast, CPAM lesions characterized by obstructed or malformed terminal bronchioles do not receive inhaled ICG and therefore remain non-fluorescent. Whereas intravenous ICG relies on differences in blood perfusion, inhaled ICG relies on differences in the ventilation. This ventilation-based contrast may provide a demarcation that closely corresponds to the true pathological boundaries of a lesion.

### Indocyanine green fluorescence in esophageal diseases

#### Identification of the esophageal pouch

The initial challenge in the surgical management of complex esophageal anomalies is the precise anatomical localization of the target structures. A systematic review by Castrillo et al. [13] described alternative applications, including intraluminal administration of ICG (0.15 mg/kg) via gastrostomy to identify the distal esophageal pouch during dissection. Zhao et al. [27] reported intraluminal administration of ICG (0.25 ml) via esophagoscopy to identify the proximal esophageal pouch. These endoluminal markings act as a fluorescent roadmap, allowing surgeons to navigate through dense connective tissues and reduce the risk of accidental injury to adjacent structures.

#### Perfusion assessment of the esophageal ends and anastomosis

Following successful mobilization, the focus shifted to ensuring the viability of the reconstructed segments. ICG fluorescence imaging is primarily used for intraoperative perfusion assessment of the esophageal ends and anastomosis in long-gap esophageal atresia (LGEA). Onishi et al. [28] reported the use of intravenous ICG (0.5 mg/kg) following the completion of delayed anastomosis after internal traction. NIR imaging allowed the visualization of perfusion in both the proximal and distal esophageal segments, confirming tissue viability. This is particularly relevant in LGEA, where prolonged traction and mobilization may compromise the vascular supply and increase the risk of anastomotic leakage or strictures.

Tsuruno et al. [29] also administered intravenous ICG (0.5 mg/kg) after completing esophageal anastomosis. Fluorescence imaging demonstrated sequential enhancement of the proximal and distal esophagus, reflecting differences in vascular supply. Notably, no fluorescence signal was observed at the anastomotic site, and no postoperative leakage occurred, suggesting that the absence of fluorescence does not necessarily predict ischemic failure.

Similarly, Castrillo et al. [13] reported the use of a lower dose (0.15 mg/kg) in five cases, achieving adequate visualization of esophageal perfusion without postoperative leakage, although some patients developed anastomotic stricture. These findings underscore that ICG provides qualitative rather than quantitative perfusion assessment, and that the current evidence does not establish definitive thresholds for predicting the outcomes.

#### Identification of H-type tracheoesophageal fistula

The precision of ICG fluorescence imaging is particularly advantageous for the intraoperative localization of small occult lesions, such as H-type tracheoesophageal fistulae (H-TEF). ICG fluorescence imaging in H-TEF is primarily used for the intraoperative localization of the fistula tract. In a case series reported by Guo et al. [30], after the induction of general anesthesia, ICG at a concentration of 0.1 mg/mL (0.1–0.3 mL) was injected directly into the fistula lumen under bronchoscopic guidance. Subsequent NIR thoracoscopic visualization enabled the clear identification of the fistula, facilitating precise clipping and division. This technique represents an endoluminal marking strategy rather than a perfusion assessment, allowing real-time anatomical navigation in a challenging surgical field where the fistula is often small and embedded within the mediastinal structures. The reported outcomes demonstrated favorable surgical performance, including short operative times, minimal blood loss, and absence of recurrence or stricture during follow-up.

However, the effectiveness of ICG in H-TEF is inconsistent. In a systematic review by Castrillo et al. [13], one case involving the endoscopic catheter-based intraluminal injection of ICG (0.15 mg/kg) failed to demonstrate intraoperative fluorescence. The authors suggested that the catheter might obstruct fluorescence transmission, thereby limiting visualization. Subsequent NIR visualization facilitates a ‘pinpoint’ approach, enabling accurate clipping and division while minimizing operative time and blood loss. However, the success of ICG-guided localization depends not only on the dose and concentration but also on the delivery technique and fistula morphology, highlighting the critical need for optimized and standardized protocols.

#### Identification of recurrent tracheoesophageal and esophagopulmonary fistulas

The clinical value of ICG fluorescence is most pronounced in the reoperative setting, particularly for recurrent tracheoesophageal fistulas (rTEF) and esophagopulmonary fistulas (EPF), where it serves as a crucial localization adjunct amid distorted anatomy and dense adhesions. Hua et al. [31] described a stepwise intraoperative localization strategy: bronchoscopy was first performed under general anesthesia to identify the fistula, followed by direct spraying of ICG into the fistulous opening. Esophagoscopy was performed if visualization was not possible. In refractory cases, ICG was applied near the previous anastomotic sites. NIR thoracoscopy allows visualization of fluorescent signals to guide dissection. The localization group demonstrated significantly reduced operative time (2.5 h vs. 3.0 h) and shorter postoperative hospital stay in patients with TEF. Notably, in EPF cases, the operative time was markedly shorter in the ICG group (2.0 h vs. 3.3 h), suggesting a particularly strong benefit in this subgroup. These findings indicate that ICG fluorescence contributes to enhanced surgical precision and efficiency; however, the standardized dosing protocols remain undefined. Although primarily used for esophageal pathologies, ICG’s high sensitivity of ICG in detecting fistulous tracts also extends to other complex thoracic conditions, especially where identifying subtle fluid communication remains a diagnostic challenge.

### Identification of the pleuroperitoneal communication

The high-sensitivity detection of fistulous tracts by ICG extends beyond esophageal pathologies to other complex thoracic conditions, with the identification of pleuroperitoneal communication (PPC) serving as an essential example of this technique. Among non-esophageal diseases, PPC is one of the most notable applications of ICG fluorescence imaging. Mori et al. [32] reported two cases of infants in which ICG (0.5 mg/kg/L) was mixed with peritoneal dialysis fluid and injected into the abdominal cavity. Under thoracoscopic observation, NIR imaging revealed fluorescent leakage through diaphragmatic defects, enabling precise identification of the communication site. Importantly, in these cases, conventional dye visualization was insufficient, whereas ICG fluorescence allowed the detection of subtle leakage that was not visible under the white light. The identified defects were successfully repaired, and peritoneal dialysis was resumed without complications. This application demonstrated that ICG fluorescence is particularly valuable for detecting minute fluid leakage pathways, offering higher sensitivity than traditional dye-based approaches. However, the evidence remains limited to small case series, and dosing strategies have not yet been standardized.

### Indocyanine green fluorescence imaging for thoracic tumors in pediatric surgical oncology

#### Indocyanine green fluorescence imaging for pulmonary metastases of hepatoblastoma

Among the thoracic applications of ICG fluorescence imaging in pediatric oncology, its role in the pulmonary metastases of hepatoblastoma (HB) is the best established. This is primarily due to the unique pharmacological properties of HB cells, which actively take up ICG and exhibit impaired biliary excretion, similar to their cells of origin in the liver. Consequently, HB-derived metastatic deposits retain ICG for a prolonged period, whereas normal lung parenchyma does not, creating a favorable tumor-to-background contrast during near-infrared imaging. This property enables ICG to function as an adjunct for the intraoperative localization of pulmonary nodules, particularly in minimally invasive surgeries, where manual palpation is limited.

In the largest cohort in the reviewed literature, Lake et al. [33] reported that among 120 resected thoracic specimens from 21 patients, ICG had a sensitivity of 91% and specificity of 57% for metastatic HB. Notably, in 10% of operations, HB-positive lesions were resected solely because of ICG avidity, and in another 40% of cases, ICG aided the localization of lesions already known from preoperative imaging; without this adjunct, these lesions may have gone undetected. Sensitivity differed according to the operative approach, reaching 95% during thoracotomy and 74% during thoracoscopic surgery. These findings indicate that ICG is particularly valuable as a highly sensitive adjunct, although false-positive fluorescence remains a limitation of this technique. These false-positive results typically occur in areas with hemorrhage, inflammation, or blood vessel irregularity, likely reflecting ICG’s binding of ICG to serum proteins and its escape from capillaries. Furthermore, limited tissue penetration is a significant technical constraint, as ICG avidity is difficult to detect if a lesion is situated greater than 1.0 cm from the parenchymal surface. Thus, while ICG significantly improved lesion localization, the results must be interpreted with caution because of these biological and physical limitations.

Delgado-Miguel et al. [34] further illustrated the practical value of ICG in thoracoscopic management of multiple metachronous HB lung metastases. In that report, ICG was administered intravenously at 0.5 mg/kg 24 h before surgery, and fluorescence-guided thoracoscopy enabled repeated limited pulmonary resections with the preservation of a functional lung parenchyma. Across four thoracoscopic procedures, 16 pulmonary lesions, including nodules as small as 1.5 mm, were resected without postoperative complications, and the hospital stay remained under 3 days after each intervention. The authors emphasize that this strategy is especially useful in the setting of multiple and/or metachronous metastases, where the lower surgical burden of thoracoscopy may facilitate repeated interventions while maintaining the respiratory function.

The principal limitation of ICG fluorescence is its restricted tissue penetration, which is generally approximately 5–10 mm from the parenchymal surface. This indicates that deeply situated lesions may have been missed during the examination. However, because HB pulmonary metastases are frequently peripheral, this limitation may be less clinically restrictive than that of other tumor types. Delgado-Miguel et al. [34] noted that the smallest lesion detected in their case measured 1.5 mm, suggesting that submillimetric foci may still escape detection. However, Kitagawa et al. [35] demonstrated a significantly higher detection threshold, identifying pulmonary metastases with diameters as small as 0.062 mm. This highlights the potential of ICG fluorescence for visualizing microscopic lesions beyond the limits of preoperative CT or manual palpations. Additional limitations include fluorescence in inflammatory or otherwise non-malignant tissues, which lowers specificity. Thus, in current practice, ICG should not be regarded as a replacement for preoperative CT, intraoperative inspection, or surgical judgment, but rather as a complementary navigation modality that improves lesion localization and may broaden the indications for thoracoscopic metastasectomy.

Whitlock et al. [37] also supported this concept in their pediatric liver cancer series, in which all patients received 0.2–0.75 mg/kg of dexamethasone intravenously 24–96 h before surgery. In their thoracic subset, ICG guidance enabled the visualization of pulmonary nodules not identified on preoperative imaging, although the reported pulmonary sensitivity was 74%, and false-negative lesions persisted. Therefore, this study reinforces the value and current technical limitations of ICG-based detection of thoracic HB.

#### Indocyanine green fluorescence imaging for metastatic pulmonary tumors other than hepatoblastoma

For metastatic pulmonary tumors other than HB, the reviewed literature centered primarily on osteosarcoma, with an additional case report on Ewing’s sarcoma treated with inhaled ICG. Compared to HB, the evidence base is still limited, and the biological basis of fluorescence appears more heterogeneous, likely relying on the enhanced permeability and retention (EPR) effect rather than an active cellular uptake.

Jeremiasse et al. [38] evaluated fluorescence-guided surgery for pulmonary osteosarcoma metastasectomy in nine pediatric patients. All patients received intravenous ICG 24 h preoperatively, initially at 0.5 mg/kg and later at 1.0 mg/kg to determine whether a higher dose improved fluorescence intensity. Intraoperatively, five of nine patients (56%) had fluorescent metastases, whereas four (44%) did not have fluorescent metastases. Among the nonfluorescent cases, two had necrotic metastases, and in two others, the lesions were likely too deep for reliable detection. Importantly, all viable metastases were fluorescent ex vivo, whereas necrotic specimens were not, suggesting that the ICG signal may correlate more strongly with viable tumors than with treated or avital deposits. No difference in the tumor-to-background ratio was found between the 0.5 mg/kg and 1.0 mg/kg groups, and the authors concluded that 0.5 mg/kg given 24 h before surgery appears to be sufficient for this indication.

The same study concluded that fluorescence-guided surgery with ICG is feasible in pediatric pulmonary osteosarcoma but should still be considered adjunctive rather than substitutive because the method cannot yet replace standard localization techniques. This is particularly relevant in thoracoscopic surgery, where the lack of tactile feedback makes any real-time visual aid potentially valuable. However, the authors explicitly stated that the additional value of ICG in intraoperative guidance requires further investigation in larger studies.

A separate technical report by Kodikara et al. [39] described the thoracoscopic resection of a subpleural osteosarcoma lung metastasis after systemic ICG administration at 4.5 mg/kg infused over 60 min, 24 h before surgery. The lesion was neither visible nor palpable under white light thoracoscopy but it was identifiable as a faint fluorescent signal under near-infrared imaging. This case suggests that ICG may be particularly useful for subpleural lesions, where the limited tissue depth allows the fluorescent signal to reach the detector to be detected. However, because this was a single case using a substantially higher dose than other pediatric reports, its findings should be interpreted as technical proof of concept rather than dose-setting evidence.

Fernández et al. [40] reported an unusual approach involving ICG inhalation. In a case report on pulmonary metastasectomy after recurrent Ewing sarcoma, ICG was not administered by intravenous administration, it was nebulized through the endotracheal tube at 0.2 mg/kg for 5 min before surgery [40]. In this negative-contrast technique, the normal ventilated lung tissue fluoresces, whereas the metastatic nodules appear as nonfluorescent defects. Conceptually, this method differs from conventional enhanced permeability and retention-based systemic fluorescence approaches and may be particularly attractive for superficial pulmonary nodules. However, as the evidence currently consists of isolated case reports, its broader oncological utility remains uncertain.

Harris et al. [41] reported a novel technique that combines intraoperative electromagnetic navigation bronchoscopy (IENB) and ICG fluorescence imaging to localize peripheral lung lesions in pediatric oncology patients. In this technique, a navigation system is used to guide the patient to the target lesion based on preoperative computed tomography (CT) images, and the lesion is marked by injecting ICG (approximately 0.5 mL) upon arrival. Subsequently, near-infrared fluorescence imaging was performed during thoracoscopic surgery (VATS) to accurately identify the lesion. This study reported successful localization in all cases, including lesions as small as 3 mm, with no complications noted.

#### Indocyanine green fluorescence imaging for thoracic tumors other than pulmonary tumors

For other thoracic tumor sites, the most informative evidence in the uploaded literature comes from Whitlock et al. [37], who examined the pathological correlation between near-infrared ICG-guided surgery and pediatric liver cancer. Their series demonstrated that ICG could assist not only with pulmonary metastases, but also with extrapulmonary thoracic disease, including lesions involving the diaphragm, chest wall, pericardium, and thoracic lymph nodes. In one patient with relapsed HB, ICG-guided thoracoscopy enabled the identification and excision of a diaphragmatic recurrence and metastatic chest wall lesion. The authors specifically noted that ICG helped delineate the lesion margins, thereby supporting gross total resection. They also illustrated pericardial and diaphragmatic diseases in the thoracic operative field, with adjacent structures, such as the phrenic nerve, identifiable in relation to the fluorescent lesions. These observations suggest that ICG may be useful not only for point localization of nodules but also for defining the disease extent along the thoracic soft tissues and serosal surfaces. Simultaneously, Whitlock et al. showed that the ICG performance for extrahepatic lesions was imperfect. Their data table reported a sensitivity of 0.74 and specificity of 0.83 for lung lesions, whereas the broader extrahepatic category showed limited specificity.

Nakamura et al. [42] described two cases of mediastinal non-seminomatous germ cell tumors (NSGCT) resected using robot-assisted thoracoscopic surgery after induction chemotherapy. In this report, the main utility of ICG was not tumor fluorescence per se, but rather the identification and preservation of the phrenic nerve in an inflamed surgical field. In one case, severe inflammation near the pulmonary hilum made it difficult to identify the right phrenic nerve, and ICG fluorescence imaging was helpful for visualizing and preserving the nerve. Therefore, this report positions ICG in mediastinal oncology surgery as a tool for functional anatomy-guided dissection rather than a direct fluorescence label for mediastinal tumor tissue.

ICG fluorescence angiography has been reported to be useful in sentinel lymph node biopsy (SLNB) for soft tissue and skin tumors in the thoracic wall. Schermerhorn et al. [43] analyzed 84 samples from 40 SLNBs, of which 81 were lymph nodes, and metastasis was found in six (approximately 7%). ICG fluorescence was positive in 76% of the lymph nodes, with a sensitivity of 79.5%, specificity of 33%, and positive predictive value of 96%. Notably, all lymph nodes that were pathologically positive for metastasis were ICG-positive, demonstrating the usefulness of ICG in detecting metastasis. Another report by Johnston et al. [44] also showed that ICG has a sensitivity of approximately 84–87%, which is higher than that of conventional blue dye. Campwala et al. [45] also reported that ICG more reliably identified radioactive sentinel lymph nodes (SLNs) than blue dyes (mean 100% vs. 78.3 ± 8.3%, *p* = 0.03).

## Discussion

ICG fluorescence imaging is rapidly developing as an intraoperative navigation technique and plays an increasingly important role in pediatric thoracic surgery. Its main uses can be broadly categorized as (1) visualization of anatomical structures (identification of luminal organs, fistulas, and lymphatic flow), (2) evaluation of the blood flow distribution and ischemia, (3) uptake by tumor cells, and (4) visualization of lesions by inhalation into normal lung tissue. Traditionally, the recognition of these anatomical features, assessment of ischemia, and diagnosis of tumor location relied on macroscopic findings and tactile sensation. However, ICG enables real-time and objective visualization, contributing to improved accuracy of intraoperative diagnosis.

However, this method has some limitations. First, the dosage, route, and timing of administration were not standardized across studies. Various methods, including lymphangiography, intravenous administration, and inhalation, have been used, making inter-institutional reproducibility challenging. For example, Takeda et al. [46] reported that ICG injected into the lungs can be distributed beyond the interlobar plane and flow into the mediastinum. Therefore, when performing lung contrast imaging, it is necessary to consider the optimal timing of imaging, as the boundaries of the contrast-enhanced area become less clear over time. Second, the fluorescence evaluation was qualitative, and an objective threshold has not yet been established. The interpretation of fluorescence intensity and arrival time depends on the operator, limiting its reliability as a prognostic indicator. Third, the limitations of near-infrared light penetration make the detection of deep lesions difficult, and fluorescence attenuation over time is also problematic.

Future prospects include the establishment of optimal protocols for each disease. Standardizing dosage and administration timing is expected to improve reproducibility across multiple institutions and contribute to the construction of evidence. This review broadly covers ICG applications in the pediatric thoracic region, from case reports to large-scale studies, and is important for understanding current trends.

Although the incidence of complications from ICG is low (0.05%–0.2%) and its severity is limited [47], allergic and anaphylactic reactions have been reported; therefore, ICG should be used cautiously in patients with iodine allergies [48]. According to the product guidelines of certified ICG distributors [12, 49], the maximum daily dose is set low for children, and safety studies should be continued for adults and the elderly, up to 5 mg/kg body weight. Adolescents (11–18 years): up to 5 mg/kg. Children (2–11 years): Up to 2.5 mg/kg. Infants (0–2 years): Up to 1.25 mg/kg.

To investigate the use, effectiveness, and safety of ICGs in the pediatric thoracic region, it is essential to accumulate data from multiple institutions because of the low incidence of these diseases. For example, Japan has the National Clinical Database, a nationwide surgical database that includes all pediatric cases [50]. If such a database can be used to collect additional information on ICGs, research has the potential to advance dramatically.

Second, improvements are expected owing to technological advancements, such as the introduction of new technologies that allow fluorescence only at target sites through fusion with molecularly targeted drugs and the use of radioisotopes that can navigate deep tissues [16]. Cytalux (pafolacianine) is a novel drug that targets folate receptors (α and β). Lehane et al. [51] reported that fluorescence-guided lung metastasis resection after pafolacianine administration was successfully performed in five young adult patients with metastatic osteosarcoma or Ewing’s sarcoma. Radioguided surgery (RGS) offers the complementary advantage of detecting signals in tissues up to 4–5 cm in diameter [16]. Once the lesion is identified, FGS provides visual cues for boundary identification and differentiation from normal tissue, facilitating a more complete and safer resection. Owing to these complementary advantages, a dual-labeling agent that enables both RGS and FGS is currently being developed [52].

Overall, ICG fluorescence imaging is a promising technique that can dramatically improve intraoperative visualization capabilities in pediatric thoracic surgery, particularly contributing to the development of minimally invasive and function-preserving surgeries. However, it remains a supplementary tool, and future challenges include protocol standardization and the establishment of evidence through large-scale prospective studies.
